# Effect of *Phaleria macrocarpa* on sexual function of rats

**Published:** 2013

**Authors:** Saadat Parhizkar, Che Zairieha Binti Che Zainudin, Mohammad Aziz Dollah

**Affiliations:** 1 *Medicinal Plants Research Centre, Yasuj University of Medical Sciences (YUMS), Yasuj, Iran*; 2 *Biomedical Department, Faculty of Medicine and Health Sciences, University Putra Malaysia, Malaysia*

**Keywords:** Andropause, Llibido, *Phaleria macrocarpa*, Testosterone

## Abstract

**Objectives:** The purpose of current study was to determine the effect of *Phaleria macrocarpa* (*P. macrocarpa*) fruits aqueous extract on reproductive performance of adult male rats by assessing the serum testosterone level and evaluating their libido behavior.

**Methods and Materials**: Eighteen male adult Spraque Dawley rats were divided into three groups and designated as treatment (240 mg/kg *P. macrocarpa* aqueous extract), negative control (distilled water), and positive control (4 mg/kg testosterone) which were supplemented through intragastric gavage for seven weeks. On the seventh week of supplementation, each of the male rats was introduced to five female rats at five different days to allow mating and observed the libido behavior. The mounting latency and mounting frequency were recorded for each mating.

**Results:** *P. macrocarpa* aqueous extract significantly increased (p<0.05) the serum testosterone level and mounting frequency of male rats. However, there was no significant effect on mounting latency. Body weight was significantly lower in rats supplemented with *P. macrocarpa* aqueous extract compared with the control groups (p<0.05).

**Conclusion:** *P. macrocarpa* showed potential value as an alternative for improving the sexual strength by increasing the level of testosterone and libido behavior. Thus, it is suggested that *P. macrocarpa* can improve the fertility in man.

## Introduction

Andropause is an age-related decline of testosterone in men associated with decrease in sperm production, libido behavior, and sexual function (Harvey et al., 2009 ▶). U.S. Food and Drug Administration (FDA) estimates that four to five million American men may suffer from low testosterone, but only 5% of them are currently being treated (Willingham, 2007 ▶). In Malaysia, 13% of men have untreatable sterility, 11% have treatable conditions, and 76% have disorders of sperm production (Concept Fertility Centre, 2006 ▶). Testosterone Replacement Therapy (TRT) is the available treatment for this problem. However, this treatment gives various side effect such as liver toxicity, benign prostatic hyperplasia, and prostate cancer (Rhoden and Morgentaler, 2004 ▶). Alternatively, plant-derived medications are believed to be safer and effective to overcome this problem. 

**Figure 1:**
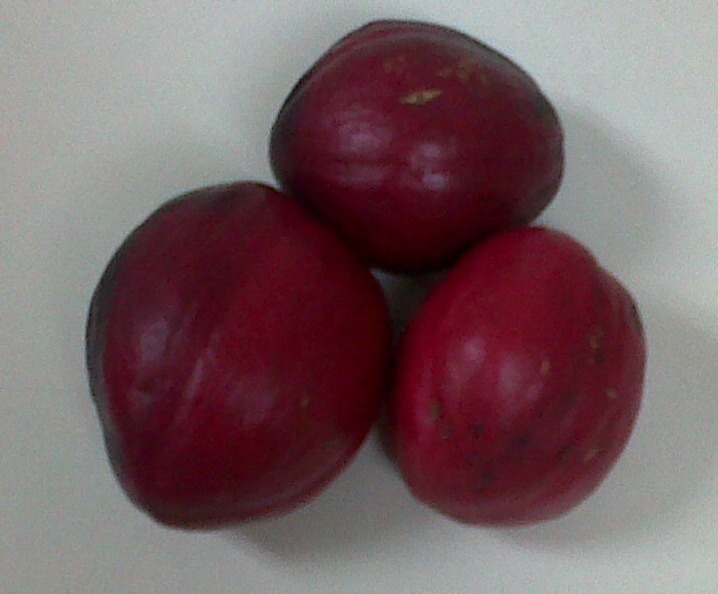
*P. macrocarpa* fresh fruit

**Figure 2:**
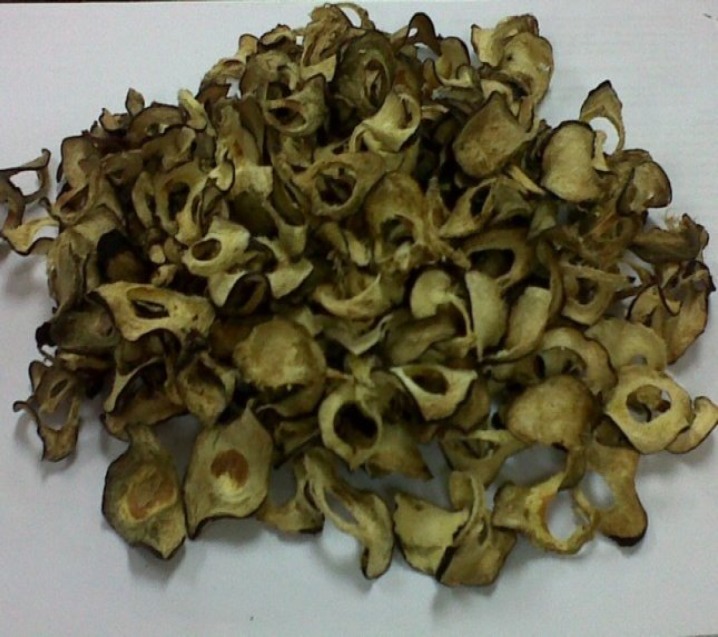
*P. macrocarpa* dried fruit slices

*P. macrocarpa* (Mahkota Dewa*) *is a type of plant that belongs to the *Thymelaeaceae *family and is commonly known as crown of god (Hendra et al., 2011 ▶). It originates from Papua Island, Indonesia and grows in tropical areas. This plant is one of the most popular medicinal plants in Indonesia (Izzati, 2010 ▶). The color of the fruit is green before ripening and red when fully ripe (Backer and van den Brink, 1965 ▶) (Figures 1 and 2). Traditionally, P. macrocarpa has been used to control cancer, impotency, hemorrhoids, diabetes mellitus, allergies, liver and heart disease, kidney disorders, blood diseases, acne, stroke, migraine, and various skin diseases (Zhang et al., 2006 ▶).

*P. macrocarpa* is believed to induce the increase of testosterone level in body. It has been claimed to improve sexual strength and libido behavior in men by the rural people. Unfortunately, there is very little published information on the potential of *P. macrocarpa* in improving male fertility. Thus, this study was conducted to exploit the potential medicinal value of *P. macrocarpa* as an attractive alternative to the synthetic hormonal drugs that are currently used to improve male fertility. Therefore, the current study was conducted to determine the effect of *P. macrocarpa* fruits aqueous extract on reproductive performance of adult male rats by determining the serum testosterone level and libido behavior. 

## Materials and Methods

**Extraction of** ***Phaleria macrocarpa***

*Phaleria macrocarpa* (Voucher no. SK1929/11) fresh fruits were supplied by Associate Prof. Dr. Mohammad Aziz bin Dollah. Two hundred and fifty grams of dried Phalera macrocarpa fruits slices were soaked in 4 L of boiled hot water until the water became half. After that, the mixture solution was filtered and the filtrate was centrifuged at 3000 rpm for 15 minutes. The supernatant was freeze-dried to obtain crystal or powder form of the extract. The powder of the extract was weighted and kept in the freezer at -20 ºC for later use. A voucher specimen (SK1929/11) was preserved at Herbarium of IBS, UPM. Sliced and air-dried *P. macrocarpa* fruits were boiled, paper-filtered, and freeze-dried. The yield obtained was approximately 13%. The extraction process was repeated till about 3 kg of dried fruit slices was extracted. 

**Working solutions**

There were three treatment groups in this study: negative control, positive control, and supplemented with aqueous extract of *P. macrocarpa*. In negative control, distilled water was use as supplement and in positive control commercial testosterone drug (Andriol® Testocap™) was used as supplement. The *P. macrocarpa* supplemented groups was given 240 mg/kg of aqueous extract of *P. macrocarpa.* The *P. macrocarpa* extract was weighted using electronic balance (AND GF3000) and reconstituted in distilled water. While the working solution for the commercial drug was used directly from the original product purchased from Schering-plough Sdn. Bhd. All of the working solutions were kept at -4 ºC. The working solutions were prepared once a week to prevent any deactivation of the active compound in the extract and to maintain the quality of the working solution.

**Experimental animals**

Eighteen Sprague Dawley male rats and ninety Sprague Dawley female rats with body weight 250-300 g, and two months old were used. They were kept in the animal house of Faculty Medicine and Health Sciences, University Putra Malaysia, under room temperature (29-32 ºC), with 70-80% humidity, and automatic 12 hours light-dark cycle. The rats were evaluated to be free from diseases and deformities. The rats were acclimatized for one week before starting treatments. They were group fed with pellet and drinking water was given *ad libitum*. 

**Experimental design**

Randomized experimental design with 3 supplementations was used for this study. The rats in each group were force fed through intragastric gavage with working solution according to their treatment groups (distilled water, 240 mg/kg *P. macrocarpa* extract, Andriol® Testocap™) for seven weeks.

**Serum testosterone analysis**

Blood samples were collected directly from the heart by cardiac puncture. All samples were collected in the morning following an overnight fast. The serum testosterone levels were assayed by radioimmunoassay technique using the kit TESTO-CTK with no. P3093 supplied by DiaSorin Diagnostics GmbH, USA. The analysis was performed using COBRA II auto-gamma analyzer.

**Libido behavior test**

On the seventh week of the treatment, female rats were introduced to the male rat for mating. They were kept in the same cage for overnight to allow mating. The female rat was first brought into on heat (oestrus) by exposing them with the bedding from the male cages four days before the mating test. This is because they allow mating only during estrous phase. The libido behavior was observed. 

**Mounting latency**

Mounting latency is the time from the entrance of female into the cage of male up to the first mounting. The time taken by the male rat to the first mount on female rat was recorded.

**Mounting frequency**

Mounting frequency is the number of mounts of male rat to female rat within 1 hour period. The number of mounts was recorded.

**Statistical analysis**

Data analysis was performed using Statistical Analysis System (SAS) version 9.2. Data of body weight, serum testosterone, mounting latency, and mounting frequency were subjected to analysis of variance (ANOVA) to analyze the significant treatment effect and the mean values between groups were compared using Duncan Multiple Range Test if F value was significant at p<0.05.

## Results

**Body weight**

**Figure 3:**
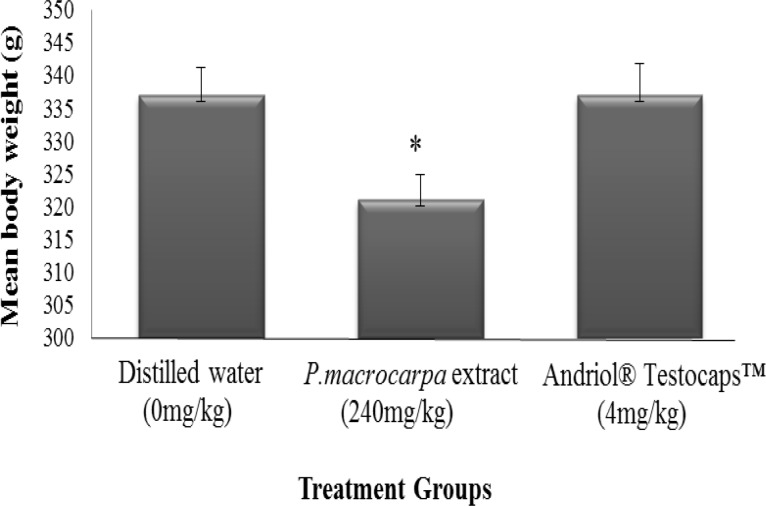
Mean of rats’ body weight supplemented with *P. macrocarpa* aqueous extract and control groups for 6 weeks. *: indicated significant differences with control group at p<0.05

The means of rats’ body weight supplemented with aqueous extract of *P. macrocarpa* for 6 weeks period are illustrated graphically in Figure 3. The mean values of body weights of the negative control, *P. macrocarpa,* and positive control groups before the study were 344, 317, and 310 g, respectively, while at the end of the study, their mean body weights were 341, 321, and 342 g, respectively. The ANOVA showed that the body weight was significantly affected (p<0.05) by the treatment. The mean of body weight for the rats supplemented with *P. macrocarpa* aqueous extract was significantly lower (p<0.05) than the control groups. 

**Serum testosterone level**

**Figure 4:**
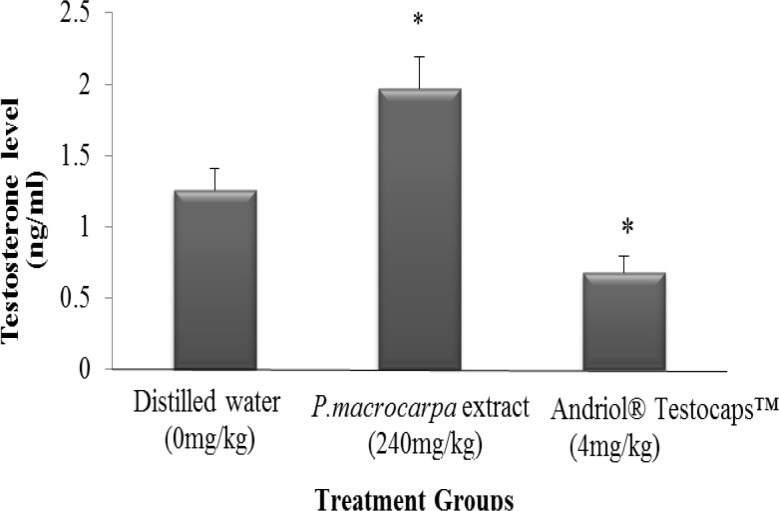
Mean values of serum testosterone level in rats supplemented with *P. macrocarpa* aqueous extract and control groups. *: indicated significant differences with control group at p<0.05

The result for the serum testosterone level was significantly different (p<0.05) between all the three treatment groups. *P. macrocarpa* aqueous extract was significantly increased the testosterone level compared with the control groups. The mean values of testosterone level of rats treated with *P. macrocarpa* aqueous extract, distilled water, and testosterone hormone group were 1.92, 1.26, and 0.68 ng/mL, respectively and are graphically illustrated in Figure 4.

**Mounting latency**

**Figure 5:**
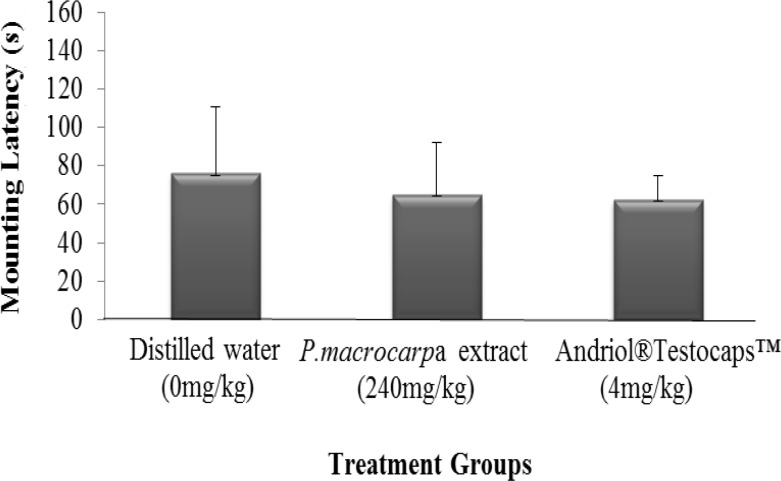
Mean values of mounting latency of rats supplemented with *P. macrocarpa* aqueous extract and control groups

*P. macrocarpa* aqueous extract did not affect the mounting latency significantly. There were no significant different between the treatment groups as shown in Figure 5. The mean values for all of the three treatment groups were ranged from 62 to 76 s. 

**Mounting frequency**

**Figure 6:**
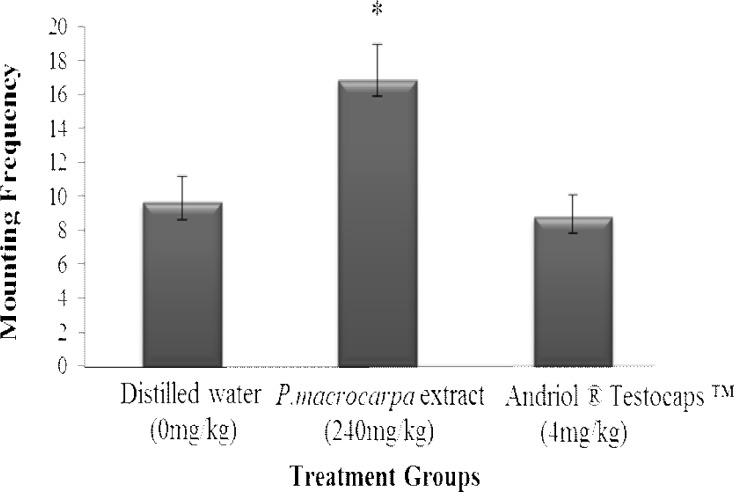
Mean values of mounting frequency of rats supplemented with *P. macrocarpa* aqueous extract and control groups. *: indicated significant differences with control group at p<0.05

Mounting frequency in this study was significantly different (p<0.05) between the treatment groups. Rats that were supplemented with *P. macrocarpa* aqueous extract showed higher mean value compared with the control groups as shown graphically in Figure 6. The mean values were 9.67, 16.89, and 8.83 for the negative control, supplemented with *P. macrocarpa* aqueous extract, and positive control, respectively.

## Discussion

The results of this study showed that the oral administration of 240 mg/kg of *P. macrocarpa* aqueous extract for seven weeks in male adult rats led to the improvement of the fertility as evidenced by significant increment of serum testosterone level.* P. macrocarpa* aqueous extract increased the serum testosterone level possibly by stimulating androgen hormone synthesis by Leydig cells. The increasing level of testosterone hormone plays an important role in increasing sexual desire and libido behavior. *P. macrocarpa* contains alkaloids and phenols which could stimulate the testosterone secretion (Al-Sa'aidi et al., 2009 ▶). In addition, *P. macrocarpa *also contain saponins that enhance androgen production. Both alkaloids and saponins exhibit aphrodisiac activity either by increasing the biosynthesis and secretion of androgens or act directly on the central nervous system to modulate the action of neurotransmitters and gonadal tissues in man (Gauthaman et al., 2002 ▶). 

The elevation of testosterone level in this study may be mediated by serotoninergic action of serotoninergic agents to enhance testosterone synthesis (Yang et al., 2004 ▶). *P. macrocarpa* aqueous extract possesses significant sexual function enhancing activity as observed in libido behavior tests. It led to significant increase in the mounting frequency as compared with control groups but it did not affect the mounting latency. 

The mounting frequency was considered as an index for both libido and potency. Therefore, this was an indication that *P. macrocarpa* aqueous extract possesses a sexual function improving effect and also an ability to increase sexual interest. The treated rats were more aggressive and were mounting continuously during the libido behavior test compared with the untreated rats. This shows both the increment of sexual performance and improvement of the libido behavior. *P. macrocarpa* aqueous extract did not affect the mounting latency and it was almost the same for all three groups. The mounting frequency indicates the sexual function improving effect when the value is lower because the reduction of mounting latency is an indication of reduction in the hesitation time of the male rats towards the receptive females. It also indicates enhanced sexual appetitive behavior because these parameters are considered to be inversely proportional to sexual motivation or desire (Yakubu et al., 2005 ▶).

This study also showed body weight reduction effect of *P. macrocarpa* aqueous extract. This was related to the lowered levels of TG and LDL and high level of HDL in the treated rats as reported by Chong et al. (2011) ▶ that *P. macrocarpa* possesses anti-hypercholesterolemic effect due to the present of gallic acid in the compound. The reduction of body weight of rats supplemented with *P. macrocarpa* also might be due to its saponin content that has stimulatory effect on testosterone hormone. Gray et al. (1979) ▶ reported that long-term treatment with high dose of testosterone reduced body weight gain and carcassed fat content. Besides that, a study by Chong, (2011) ▶ showed that *P. macrocarpa* significantly reduced body weight gain, total cholesterol, triglyceride, HDL and LDL, and up regulated hepatic LDL receptor. The reduction of the body weight of the rats that were treated with *P. macrocarpa* is due to the increase in fat metabolism in their body. *P. macrocarpa *significantly increases the fat metabolism in the body and the fat is metabolized more rapidly which contributes to the lower body weight. Alternatively, some natural products may function by simply delaying the transfer of glucose from the stomach to the small intestine, the main site of glucose absorption (delayed gastric emptying rate) (Shane-McWhorter, 2001 ▶; Yeh et al., 2003 ▶). According to Ali et al. (2013) ▶ weight gain reduction in *P. macrocarpa* -treated rats might be resulted from providing glycaemic control without hyperinsulinaemia and consequently control of body weight gain. 

*P. macrocarpa* showed potential value as an alternative for improving the sexual strength by increasing the level of testosterone and libido behavior. Thus, it is suggested that *P. macrocarpa* can possibly improve the fertility and overcome the impotency problem in men.
